# The *Pros* and *Cons* of Cystic Fibrosis (CF) Patient Use of Herbal Supplements Containing *Pulmonaria officinalis* L. Extract: the Evidence from an In Vitro Study on *Staphylococcus aureus* CF Clinical Isolates

**DOI:** 10.3390/molecules24061151

**Published:** 2019-03-22

**Authors:** Beata Sadowska, Urszula Wójcik, Justyna Krzyżanowska-Kowalczyk, Mariusz Kowalczyk, Anna Stochmal, Joanna Rywaniak, Julia Burzyńska, Barbara Różalska

**Affiliations:** 1 Department of Immunology and Infectious Biology, Institute of Microbiology, Biotechnology and Immunology, Faculty of Biology and Environmental Protection, University of Lodz, Banacha 12/16, 90-237 Lodz, Poland; beata.sadowska@biol.uni.lodz.pl (B.S.); urszula.wojcik@unilodz.eu (U.W.); joanna.rywaniak@biol.uni.lodz.pl (J.R.); j.burzynska@ipczd.pl (J.B.); 2 Department of Biochemistry, Institute of Soil Science and Plant Cultivation, State Research Institute, Czartoryskich 8, 24-100 Pulawy, Poland; jkrzyzanowska@iung.pulawy.pl (J.K.-K.); mkowalczyk@iung.pulawy.pl (M.K.); asf@iung.pulawy.pl (A.S.)

**Keywords:** lungwort fractionated phenolic extract, cystic fibrosis, *Staphylococcus aureus*, adhesion, biofilm, cytotoxicity

## Abstract

The justification for the use of herbal supplements with *Pulmonaria officinalis* L. extract (POE) in the case of staphylococcal lung colonization/infections characteristic for cystic fibrosis (CF), was examined in vitro. The impact of POE phenolic-rich fraction on the virulence attributes of CF-associated *Staphylococcus aureus* (*S. aureus*) clinical strains has been assessed, including pathogen adhesion, biofilm formation on native and protein-conditioned surfaces (mucin, elastin), mature biofilm eradication, staphylococcal protein A expression, α-toxin release, and *S. a.* adhesion to A549 cells. Cytotoxicity of the extract to lung epithelial cells was also investigated. It was found that POE has bacteriostatic effects at MIC 1–2 mg/mL, recognized as of limited efficacy, but at MIC/subMICs it targeted virulence not viability. It usually decreased *S. aureus* adhesion and less frequently inhibited biofilm formation on native and protein-conditioned surfaces. Observed effect seems to be related to significant reduction by POE of sortase A activity. However, in some cases POE favored the creation of biofilm by staphylococci and *S. aureus* adhesion to the lung epithelium was not limited. On the other side POE caused significant decrease of *S. a.* α-toxin synthesis and slightly weakened the expression of SpA. When used at supraMICs POE eradicated mature biofilm, but in some cases with unsatisfying outcomes. Promisingly, POE has been recognized as a safe product, with no cytotoxicity up to 4 mg/mL. These results reflect the positive, negative or neutral anti-staphylococcal properties of POE. It seems that POE may be beneficial as a prophylactic, but not as a therapeutic or supportive agent in the area of CF—integrative medicine. However, introduction the official recommendations needs further in vivo studies.

## 1. Introduction

The increasing reductions of antibiotics’ efficacy makes it necessary to seek new therapeutic options. Discovering new antimicrobials expressing direct biostatic/biocidal effects or modifying the pathogenic potential of microbes are equally important. In this context, plant-derived products are of interest since their health-promoting and antimicrobial properties are known [1,2]. Many of them have been commonly used in folk medicine despite a lack of precise knowledge concerning their mechanisms of action. For example, the plant *Pulmonaria officinalis* has been cultivated as perhaps one of the most widespread in Europe medicinal herbs. Following the “Doctrine of Signatures” [3], its ovate spotted leaves seemingly resembling diseased lungs give it the popular name of lungwort. The medical utility of many plants as remedies has been discredited, however, new research suggests that lungwort has certain traits, specifically as an antioxidant and secretolytic, that might benefit lung health. The most important lungwort properties include antitussive, astringent, demulcent, emollient, expectorant, mucilaginous, and tonic activities [4,5]. Its action as a demulcent to relieve irritation of mucous membranes in the upper respiratory tract by forming a protective slime-like film is very attractive. Available herbalist knowledge led us to evaluate the usefulness of POE in supporting lung infection treatment in cystic fibrosis (CF) patients.

CF is a common life-threatening autosomal recessive disease caused by mutations in the transmembrane conductance regulator gene (*CFTR*). CF affects ~1 in 4000 newborns in the US, and more frequently in some European countries [6]. Over 44,700 cases in 31 countries were reported (data from European Cystic Fibrosis Society Patient Registry 2016), and the number is growing each year. Children represent 47.6% of cases, with the proportion of adults varying between countries. The mutations in a member of the ATP-binding cassette transporter family of genes, encoding an anion transporter protein (CFTR) in the epithelium, influences chlorine and bicarbonate transport, with a secondary effect on sodium transport [7,8]. It increases salt concentration in sweat and thickened secretions in other organs. Disturbed mucocilliary clearing in the respiratory tract means that 20–65% of CF patients are chronically colonized/infected with *Pseudomonas aeruginosa*. This concerns mainly adults, because the etiology of CF-associated lung infection in children is quite different. *Haemophilus influenzae* and then *S. aureus* are pathogens most commonly isolated from infants and young children, with a peak for *S. aureus* at 11–15 years. These two pathogens infect the bronchial tree of young patients damaging the epithelium and causing chronic inflammation, which leads to the subsequent introduction and adhesion of other bacteria and fungi [6,7,8,9,10]. Similar case statistics and epidemiology profile to Europe are found in USA (U.S. Cystic Fibrosis Foundation 2016 Annual Report [10]).

The microbiome of the CF lungs is complex as multi-level interspecies interactions take place. Numerous commensals inhabiting the airways play a role in these interactions, although these roles are not yet fully understood. *S. aureus*—the species that is the subject of our research—is considered to be a commensal of the nasal mucosa and upper respiratory tract, but also it is a dangerous pathogen if is found in other parts of the body, including the lower respiratory tract. The proof is the aforementioned isolation of *S. aureus* from young patients with CF, reaching 70% of all isolates [6,7,8,9,10]. It is widely accepted, however, that infections of CF-airways are frequently polymicrobial, and a mechanism common to most CF bacterial pathogens is growth in aggregates or biofilms, which once established are rarely eradicated with conventional antimicrobials. Biofilm formation by *S. aureus* does not differ significantly from that created by other microorganisms [11,12]. Staphylococci, both free floating and sessile communities, possess a wide range of surface adhesins, including microbial surface components recognizing adhesive matrix molecules (MSCRAMMs), which bind most host extracellular matrix (ECM) proteins, e.g., fibronectin, fibrinogen, collagen, vitronectin, laminin, and elastin. The list of adhesins also comprises staphylococcal protein A (SpA), biofilm-associated protein (Bap), *S. aureus* surface protein G (SasG), extracellular adhesive protein (Eap), extracellular fibrinogen binding protein (Efb) and others [13,14,15,16]. The important role in the pathogenic process of staphylococcal invasive enzymes and some toxins, such as α-hemolysin, should also be noted [17]. In addition to the outstanding ability of staphylococci to form biofilms, structures highly resistant to the action of conventional antibiotics, attention should be paid to the risk of colonization/infection with methicillin-resistant *Staphylococcus aureus* (MRSA) which creates a clear need for new therapeutic solutions.

Currently there is a growing trend for infected CF patients to use classical medication directed against respiratory tract-associated symptoms, together with so-called integrative medicines. Herbals, among many others, may offer beneficial supportive therapy [18]. However, better understanding is required to assess their proper use. The main goal of our research is to provide evidence regarding the use of paramedical products containing lungwort extract by patients with cystic fibrosis and chronic colonization/staphylococcal infection. The novelties of the study are: firstly, the use of a fractionated and well characterized extract from the herb *P. officinalis* L.; secondly, an attempt to verify its suitability in solving a specific medical problem, studied in vitro under conditions partially simulating the microenvironment of the respiratory system.

## 2. Results and Discussion

### 2.1. Short Description of Pulmonaria Officinalis L. Fractionated Extract

The original, rich in polyphenols methanolic fraction of *Pulmonaria officinalis* L. extract (hereinafter referred to as POE), described by Krzyżanowska-Kowalczyk et al. [19] was used. The aim of the study was to validate in vitro alternative medicine recommendations about POE-containing products usage for airways disorders, including these in cystic fibrosis (CF) patients. This herb, called lungwort, has been used for centuries in folk medicine to treat chest infections as it has antimicrobial properties. Lungwort is also available today a pharmaceutical product in natural forms, such as in tinctures, teas and capsules. *Herba pulmonariae* is registered in the National Database of Health Protection Products in Poland, authorized for pharmaceutical use [5]. Some reports indicate that lungwort contains a several compounds known to possess biological effects, but its detailed composition has only recently become available. In contrast, tested POE has been fully characterized [4,19]. Its main constituents are phenolic acid derivatives, primarily conjugates of caffeic acid with danshensu—rosmarinic, monardic, lithospermic, salvianolic, shimobashiric and yunnaneic acids. Esters of caffeic acid, quinic (so-called chlorogenic acids), threonic and glyceric acids, and coumaroylquinic acids are present. A few lignans (globoidnans A and B, pulmonariosides A and B) and common flavonol glycosides (quercetin and kaempferol derivatives) were identified. The phytochemical profile from the estimated content of individual groups of specific metabolites and their quantitisation are given in Figure S1 and Tables S1 and S2 (Supplementary Materials).

Tested POE had no high biostatic activity—its MIC was 1–2 mg/mL, for both clinical and reference *S. aureus* strains, regardless of the sensitivity profile of reference strains to antibiotics. However, the potential indirect activity of POE was of our interest. Heras et al. [2] and other authors [20] have looked for products that restrict the expression of microbial virulence factors, activate host immune defenses and/or show synergistic effects with conventional antimicrobials. Unlike synthetic molecules, phytochemicals have an unmatched structural diversity with a spectrum of interactions that offers new methods for controlling drug-resistant infections. Plant extracts contain a multitude of metabolites with well-documented broad antimicrobial activity, including also substances effective against *Staphylococcus aureus*. Such properties were identified in certain phenolic acids such as: caffeic [21], 3-*p-trans*-coumaroyl-2-hydroxyquinic [22,23], rosmarinic acid [24,25], the flavonoids kaempferol and quercetin [26,27], belonging to the curcuminoids group of curcumin and its derivatives [28,29,30]. The high content of rosmarinic, caffeic, chlorogenic acids and their transformation products in POE is shown in Table S2. The mechanism of action of many phytochemicals is not understood, although these particular ingredients have been extensively investigated and knowledge of their exact antimicrobial targets is well established.

Rosmarinic acid, the main constituent of our POE (~260 µg/mg; Table S2), is probably responsible for the biological activities we observed. Slobodníková et al. [24,31] found rosmarinic acid effective not only against planktonic forms of *S. aureus,* but able also to suppress biofilm formation at concentrations near the MIC. This is a relevant observation because both lifeforms of these bacteria—the planktonic and sessile (biofilm) form—are equally important in pathogenesis. Other compounds in POE, such as quercetin and kaempferol, have anti-biofilm activity against several bacterial species including *S. aureus*, downregulating the *agr* system via anti-quorum sensing (QS) activity, consequently decreasing synthesis of hemolysins and other virulence factors [11,15,31,32,33]. However, no data on antiadhesive/anti-biofilm activity of *P. officinalis* extract is available, although numerous plant extracts were tested for activity against *S. aureus* [34].

### 2.2. POE Antiadhesive and Anti-Biofilm Properties

**Figure 1:**
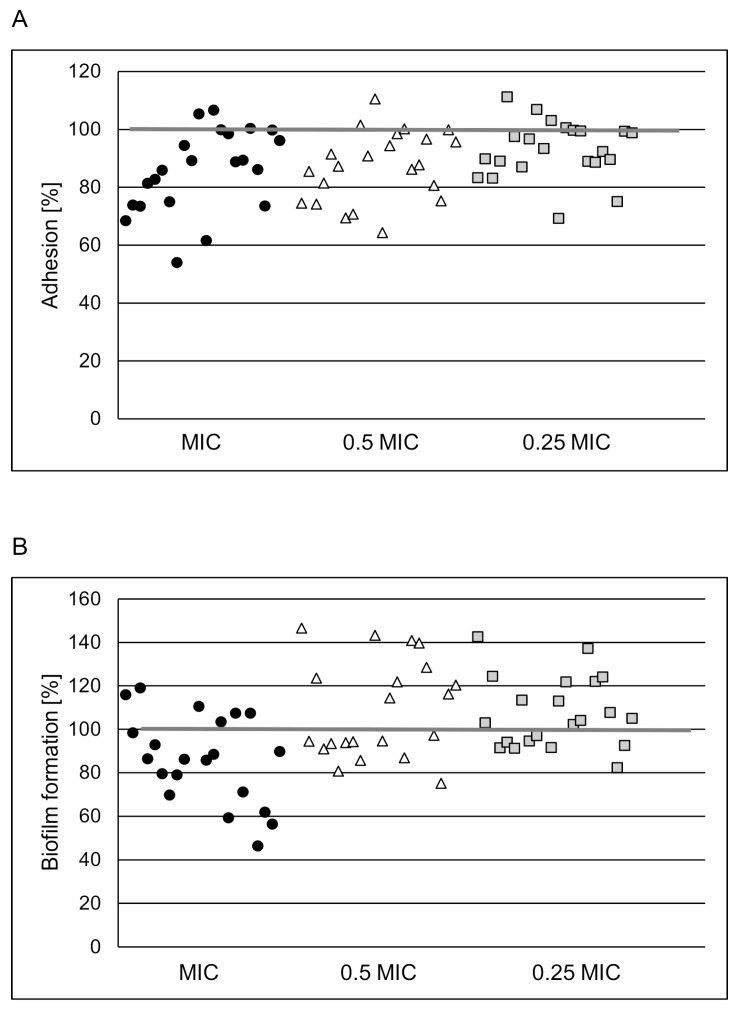
Adhesion (**A**) and biofilm formation (**B**) by *S. aureus* reference and clinical strains (n = 2 + 20) on abiotic surface in the presence of POE used at MIC (circles), 0.5 × MIC (triangles), 0.25 × MIC (squares), tested by a LIVE/DEAD BacLight Bacterial Viability Kit. The percentage of adhered cells or biofilm biomass compared with positive control (untreated bacteria) considered as 100% is presented.

Since efficient adhesion of bacteria to the surface is the first step of biofilm formation, its prevention is an important therapeutic approach. Our data shows that POE at MIC and subMICs reduced by ~54% *S. aureus* adhesion to the inert surface. The effect was strain- and POE concentration- dependent (Figure 1A). The highest applied concentration of POE (MIC) did not decrease staphylococci viability upon co-incubation for 1 h (data not shown). Thus, noticed weakening of bacterial adhesion due to POE did not result from a reduction in viability, but probably through a reduction of surface adhesin expression.

**Figure 2:**
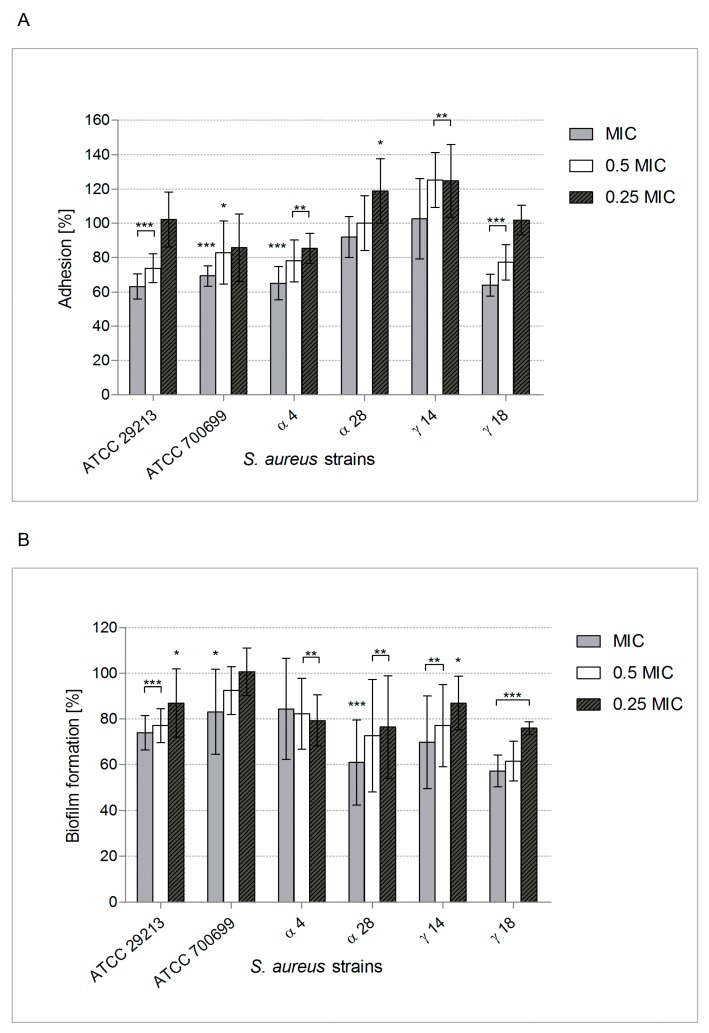
Adhesion (**A**) and biofilm formation (**B**) by *S. aureus* reference and clinical strains (n = 2 + 4) on mucin- conditioned surface, in the presence of POE used at MIC, 0.5×, 0.25 × MIC, tested by LIVE/DEAD BacLight Bacterial Viability Kit. The percentage of adhered cells or biofilm biomass compared with positive control (untreated bacteria) considered as 100% is presented. Significant differences: * *p ≤* 0.05, ** *p ≤* 0.01, *** *p* < 0.001.

**Figure 3:**
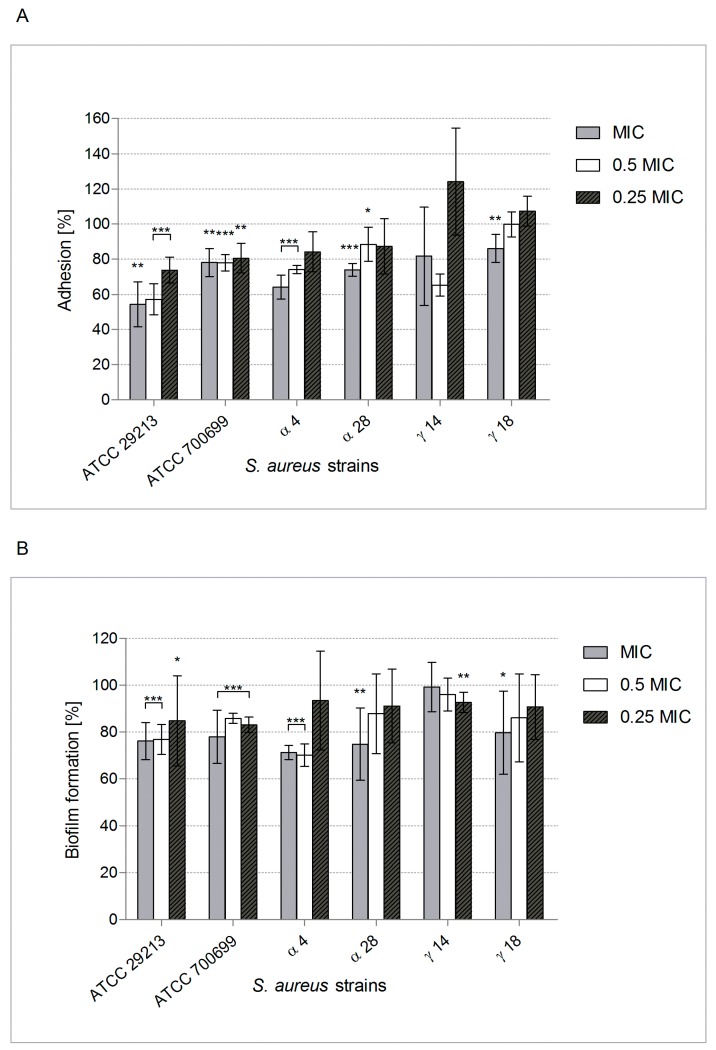
Adhesion (**A**) and biofilm formation (**B**) by *S. aureus* reference and clinical strains (n = 2 + 4) on elastin-conditioned surface, in the presence of POE used at MIC, 0.5×, 0.25 × MIC, tested by LIVE/DEAD BacLight Bacterial Viability Kit. The percentage of adhered cells or biofilm biomass compared with positive control (untreated bacteria) considered as 100% is presented. Significant differences: * *p ≤* 0.05, ** *p ≤* 0.01, *** *p* < 0.001.

This observation prompted us to test this under conditions closer to those found in vivo in CF patients with lung infection complications, with particular attention to mucin and elastin presence [6,8,10]. Therefore, the surfaces were coated with them to mimic respiratory tract mucosa in an assessment of *S. aureus* adhesion influenced by POE. Figure 2A shows that adhesion to mucin was usually decreased by 20–36% (*p* < 0.001–0.01). This significant reduction of adhesion due to POE at MIC (*p* < 0.001) and 0.5 × MIC (*p* < 0.01) was also similar in the case of elastin-conditioned surfaces. POE at MIC decreased *S. aureus* adhesion to elastin by 14–45% (Figure 3A).

Thus, POE-induced inhibition of *S. aureus* adhesion to these ligands could be advantageous for the CF patient. Our subsequent experiments showed that POE can also reduce biofilm formation by *S. aureus* on both inert and mucin- or elastin-coated surfaces (Figure 1B, Figure 2B and Figure 3B, respectively). Unfortunately, for most of the tested strains the effect was not significant with a reduction of ~20%. It is noteworthy that intensification of up to 40% in biofilm formation by *S. aureus* exposed to POE at subMICs was also observed. This phenomenon is not surprising because microorganisms under unlethal stress usually trigger efficient defense reactions (SOS), sometimes involving more effective biofilm building.

The ingredients of plant extracts with low biostatic/biocidal potential can be used by microorganisms as extra metabolic substrates. They may also be agonists or antagonists of molecules operating in QS systems, down- or up-regulating genes encoding products important in metabolic processes [1,2,31]. However, the use of subMICs of tested product during our in vitro study is justified, since it simulates the conditions encountered in real life situations. From in vivo animal models and clinical pharmacokinetic/pharmacodynamic studies it follows that in some soft tissues (e.g., in the subcutaneous layer, wounds, mucous membranes, reproductive system or respiratory tract), pathogens are often exposed to low concentrations of biocides. In asking how this occurs, first it is because plasma concentrations may fall below the MIC level by the end of the dosing interval for antibiotics/other biocides with a short half-life and only occasional administration; second, as a result of poor diffusion to the infection site, especially after oral administration as assumed for most POE-containing products; third, low plasma and tissue exposure may occur in cases of drug-drug interactions; finally, sub-MICs probably occur due to poor drug stability and after uncontrolled self-administration [12,34,35].

Regarding the use of herbal products containing POE by CF-patients suffering from chronic *S. aureus* infections, our findings are not that optimistic. POE was only marginally effective in eradicating preformed staphylococcal biofilms. Raising the POE concentration to 4 × MIC did not significantly increase this potential (eradication by ~30% for one strain and no effect for other strains, data not shown). Thus after a plausible in vivo physiological reduction in the effective concentration of metabolized phytocompounds, eradication of mature bacterial biofilm seems almost impossible. One can increase in vitro the concentration of the product to get the desired effect, but it would be impossible to achieve such concentrations in vivo without potential side effects [14].

### 2.3. POE Impact on Some of the Main Staphylococcus Aureus Virulence Attributes

Protein A (SpA) expression by *S. aureus* pre-cultured in TSB with or without POE was assessed in our study because SpA is a protective antigen presented on the surface and secreted by all *S. aureus* strains, allowing bacteria to manipulate the host immune response. Apart from SpA interfering with opsonization, it might have a direct effect on the respiratory epithelial cells, even in the absence of IgG. In respiratory tract infections, SpA induces chemokine IL-8 expression, causing recruitment of leukocytes mediated by interaction with TNF-*α* receptor widely expressed by airway epithelium [15]. SpA is also known as responsible for the aggregative phenotype of staphylococci and capacity for biofilm formation [12,16]. Performed immunofluorescence assay giving the percentage of SpA expression by *S. a.* cells exposed on POE, showed no significant reduction (<17%, data not shown). It is known that protein synthesis inhibitors decrease the activity of staphylococcal virulence factors, including SpA [35], thus POE does not seem to possess such a property. Other possible explanations include the effect of POE on membrane enzymes activity, e.g., sortases, housekeeping enzymes responsible for the correct spatial organization and expression of surface adhesive molecules and other factors involved pathogenesis, which makes them a suitable target for inhibition during bacterial infections treatment [1,31,34,36,37]. Since adhesion of *S. aureus* to an abiotic surface and surfaces modified by mucin/elastin, known to be mediated by various MSCRAMMs was impaired by POE, the above suggestion is plausible and we confirmed it in the experimental investigation. POE used at 500 µg/mL, which corresponded with 0.25 × MIC for most of tested *S. aureus* strains, strongly inhibited activity of staphylococcal sortase A (by 70.3% in comparison to positive control of fully active enzyme).

Besides numerous cell-wall associated virulence factors (adhesins), the *S. a.* armoury includes tissue-damaging enzymes and toxins affecting not only in nutrition, but destruction of host tissue and depressed immune responsiveness. Among them α-hemolysin (Hla)—pore-forming cytotoxin active against many types of cells (e.g., human platelets, monocytes and erythrocytes) and able to enhance biofilm formation, generates the most interest as potential target for new drugs [17,37].

In our study the release of Hla was significantly decreased after *S. a.* exposure on POE used at 0.5× and 0.25 × MIC—up to ~50% (Table 1, * Significant differences compared to the control (*p* ≤ 0.05). This observation is relevant and allows us to suggest that POE may be a potential novel staphylococcal virulence inhibitor. Together, our extract noticeably impaired staphylococcal adhesion, but was ineffective against biofilm formation by *S. aureus* strains, failing to eradicate preformed biofilms. Moreover, there was no inhibitory activity of the extract on *S. aureus* adhesion to A549 cells; indeed bacterial adherence to the “host cells” increased by 4–44% (*p* from 0.04 to 0.0001). Furthermore, expression of important pathogenic factor—SpA was only weakly affected in a concentration- and strain-dependent manner. However, it is worth noting the significant reduction in the production of the α-toxin and the activity of sortase A. It is worth noting, however, that *S. aureus* isolates have about 17–21 surface proteins with the LPXTG motif, the target for the activity of sortase A, which are involved in adhesion and invasion. Inhibition of SrtA activity may therefore have a different, strain-dependent effect of changing the surface expression of the modified bacterial proteins [36,37]. For example a portion of SpA is released into the supernatant with an intact sorting signal, and release of SpA was reduced when the native sorting signal of SpA was replaced with the corresponding region of another sortase-anchored protein, namely, SdrE [38].

**Figure 4:**
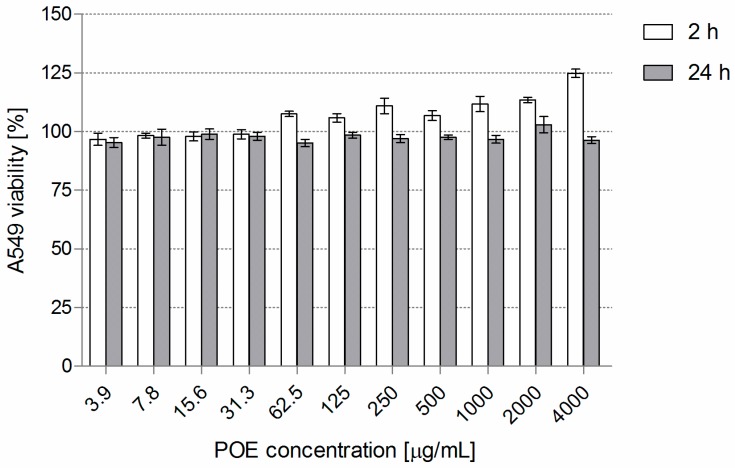
Cytotoxic effect of POE used at a concentration range of 3.9–4000 µg/mL on human lung epithelial cell line A549 (exposure time: 2 h and 24 h), tested by MTT-reduction assay. The mean cell viability (% of cell viability ± S.D.) compared with positive control (untreated cells) considered as exhibiting 100% viability is presented.

Considering the composition of POE, the results of our multidirectional research, evaluated as a whole, are unexpected and the data prompts us to undertake a more thorough analysis. Rosmarinic acid (RA), being the dominant component of the fraction, might be responsible for its positive antimicrobial activity. This assumption came from in vitro tests of this phytochemical as an antibacterial, anti-inflammatory, immunomodulating agent, etc. [27,34]. Moreover, RA seems to be an excellent example of a compound for which the bioavailability/biosafety processes can be simulated effectively in in vivo models, especially as it is well absorbed from gastrointestinal tract and the skin. After oral administration, RA is rapidly eliminated from blood circulation. However, it is metabolized predominantly to caffeic, coumaric and ferulic acids, which also possess antimicrobial potential [21,22,23,28]. RA is only weakly toxic in vitro. Indeed, our POE containing 262 µg/mg RA, used at 3.9–4000 µg/mL against airway epithelial cells (A549), was also generally recognized as safe (GRAS) since it did not reduce in vitro cell viability after 2 and 24 h co-incubation (target cell viability remained at not less than 95%; Figure 4).

The clear conclusion is that the biological activity of isolated phytochemicals does not compare with that of full plant extracts containing them, in which multidirectional interactions of numerous components occurs, including complementary and synergistic interactions that determine their biological profile of activity.

Therefore, the answer to the main question whether complex plant-derived supplements used by CF patients either arbitrarily or with the advice of a physician are fully safe (i.e., do not have of significant adverse effects on eukaryotic cells), in case of our POE is a simple positive, but routinely required cytotoxicity testing does not give a complete answer. Other functions of the host barrier cells, including the secretion of cytokines/chemokines/mediators of inflammation etc., need to be investigated. One cycle of in vitro tests cannot answer all the questions that arise. Moreover, we propose that greater impetus for a more in-depth analysis of the results is needed from in vitro studies.

Our discussion should alert the scientific community to undertaking a more critical approach to assessing the biomedical potential of non-antibiotics. The *pros* and *cons* regarding the use of the products safe because of social beliefs in the integrative therapy of patients with CF have been given. The fact that some of them—genistein, resveratrol, curcumin, etc.—have grown in popularity among CF patients and may be beneficial [18] does not mean that every natural product will have more positive than negative effects. None of the POE inhibitory effects is sufficiently substantial to consider the extract is good as a therapeutic preparation against staphylococcal infections. However, demonstrated biological activity of POE entitle us to suggest the use of POE-containing preparations in prophylaxis to prevent tissue colonization by *S. aureus*.

Anyhow, the encouraging effects of POE-containing herbal supplements probably will not always be fully confirmed. However, apart from the unsatisfactory anti-biofilm activity of POE, it is necessary to pay attention to the wider biological properties of the extract and its components, such as anti-inflammatory and anti-oxidant activities [4,5,19,20,24]. Moreover, some natural products are believed to be potentiators or correctors of mutated *CFTR* products that give relief of CF-associated dysfunctions [18]. Therefore, we are convinced that the results of further research in this direction, including in vivo study, may turn out to be promising.

## 3. Materials and Methods

### 3.1. Pulmonaria Officinalis Fractionated Extract (POE)—Preparation and Characterization

*P. officinalis* L. aerial parts were purchased from Kania (Częstochowa, Poland). Plant material was ground, defatted with chloroform and extracted twice with 80% aq. methanol. The extract was purified by SPE [19]. Polar constituents were removed with 1% methanol, whereas the phenolic fraction was eluted with 50% MeOH (*v/v*); details of procedures and identification of compounds are given in the Supplementary Materials. The stock solution of the phenolic-rich fraction of the extract (20 mg/mL) was prepared in sterile-filtered water and diluted in the liquid medium used experimentally for the required concentration range.

### 3.2. POE Cytotoxicity

Monolayers of human lung carcinoma-derived epithelial cells (A549, ATCC, CCL-185) were cultured as previously described [39]. Briefly, a detached cell suspension (5 × 10^5^ cells/mL) was seeded at 100 µL/well into 96-well tissue culture plates (Nunc, Roskilde, Denmark) for 24 h at 37 °C. The culture medium was replaced with 100 µL medium with POE (3.9–4000 µg/mL) for 2 and 24 h, with appropriate positive and negative controls. The cytotoxicity was measured by MTT-reduction assay. The final absorbance (A_550_) of the samples was assessed using a microplate reader (Victor2, Wallac, Turku, Finland), and the percentage of viable cells and the IC_50_ were calculated.

### 3.3. Bacteria

*S. aureus* ATCC 29213 (methicillin-sensitive); ATCC 700699 (methicillin-resistant/glycopeptide intermediate sensitive), and 20 clinical isolates from sputum of children (1.5–19 years) with CF and chronic respiratory tract infections were used. *S. a.* clinical strains had been extensively characterized by Sadowska et al. [40]. They were kept frozen at −80 °C in tryptic soy broth (TSB, BTL, Łódź, Poland) with 15% glycerol. The ready-to-use cultures were freshly prepared in TSB with 0.25% glucose, the suspension density being adjusted to the appropriate value for the given test.

### 3.4. POE Minimum Inhibitory Concentration (MIC)

MIC of POE against *S. a.* (n = 22) was evaluated twice using a microdilution broth assay according to EUCAST recommendations [41].

### 3.5. Impact of POE on S. a. Adherence and Biofilm Formation on Abiotic Surface and Conditioned with Mucin or Elastin

Adhesion of all *S. aureus* strains (n = 22) was tested on uncoated polystyrene 96-well microplates, and of selected strains (n = 6; ATCC 29213, ATCC 700699, α4, α28, γ14, γ18) on microplates with immobilized mucin (Roth, Karlsruhe, Germany) or elastin (Sigma, St. Louis, MO, USA). Conditions of surface “coating” were as follows: 100 μL/well mucin/elastin (10 mg/mL or 0.1 mg/mL in PBS, respectively), overnight incubation at 37 °C, removal of mucin/elastin, washing with 200 μL/well PBS, blocking with 200 μL/well 2% BSA in PBS (overnight at 4 °C), and washing with 200 μL/well PBS. Then 100 μL POE at MIC; 0.5×; 0.25 × MIC and 100 μL of *S. a.* suspension at OD = 0.9 (~5 × 10^7^ CFU/mL), and incubation for 1 h at 37 °C (variant A = adhesion). Biofilm formation was evaluated after 24 h (variant B = biofilm). Proper negative and positive controls were included. POE effects were assessed by LIVE/DEAD BacLight Bacterial Viability Kit (Invitrogen, Molecular Probes, Eugene, OR, USA). Each variant of the experiment was run twice in quadruplicate. The results are given as the percentage of adherent bacteria/biofilm biomass calculated from the mean fluorescence values of test wells compared to the positive control (untreated bacteria; taken as 100%).

### 3.6. Anti-Biofilm Potential of POE

To investigate the effect of POE at MIC; 2×; 4 × MIC on preformed *S. a.* ATCC 29213 and clinical α28, γ14 strains-derived biofilms, the same protocol of their development as in variant B (Section 3.5) was introduced. After 24 h incubation of the biofilm at 37 °C with POE or without (control), the degree of biomass survival (%) was assessed as described above.

### 3.7. POE Influence on S. a. Protein A (SpA) Surface Expression and α-Hemolysin (Hla) Synthesis

Reference ATCC 29213, ATCC 700699, clinical α4, α28, γ14, γ18 *S.a.* strain suspensions (OD = 0.9) were cultured for 24 h at 37 °C in medium alone or with POE at 0.5 × MIC, 0.25 × MIC. Bacteria were centrifuged at 2500 rpm for 10 min and the supernatants frozen at −20 °C until α-toxin synthesis was measured. Hla was determined by a sandwich ELISA on MaxiSorp microplates (Nunc). Rabbit monoclonal anti-Hla Ab (Sigma, St. Louis, MO, USA) diluted 1:1000 in PBS were used as coating Ab (ON/+4 °C). The samples and Hla (Sigma) at a concentration range of 31.2–4000 ng/mL as a standard were added for 1.5 h at room temperature (RT). Then sheep polyclonal anti-Hla Ab (Abcam, Cambridge, UK) diluted 1:500 in PBS/1% BSA, HRP-conjugated donkey anti-sheep IgG Ab (Sigma) diluted 1:1000 in PBS/1% BSA (both 1.5 h/RT) and ready-to-use ABTS (Sigma) were used for detection of captured Hla. The concentration of Hla released to the supernatants was calculated using the standard curve of reference Hla based on absorbance measured at λ = 405/490 nm (Victor2). Two independent experiments were performed, each in duplicate. Assessment of protein A (SpA) expression in cell pellets relied on immunofluorescence assay [42], measured at 485_ex_/535_em_ nm (Victor2, Wallac, Turku, Finland). The percentage of SpA expression compared to the positive control (taken as 100%) was calculated. Two independent experiments were run in duplicate.

### 3.8. Effect of POE on Staphylococcal Sortase A (SrtA) Activity

To test effect of POE at 500 µg/mL on SrtA activity SensoLyte 520 Sortase A activity Assay Kit Fluorimetric (AnaSpec Inc. Fremont, CA, USA) was used according to the manufacturer’s instructions. The fluorescence reading from the substrate control were used as the background and subtracted from the readings of the other wells. The results are given as the percentage of SrtA activity calculated from relative fluorescence units (RFU) of test wells compared to the positive control (untreated SrtA taken as 100%).

### 3.9. S. a. Adherence to A549 Cells

Reference ATCC 29213, ATCC 700699, clinical α4, α28, γ14, γ18 *S. a.* strains (suspensions at OD = 1.8, ~1 × 10^8^ cells/mL) were labeled (fluorescein isothiocyanate; 1 mg/mL FITC, isomer I, in PBS) at RT for 20 min [40]). Semiconfluent A549 monolayers were treated as follows: labeled bacteria (100 µL) and 100 µL POE (at MIC, 0.5×; 0.25 × MIC) were added (6 repetitions) and incubated for 1 h at 37 °C, air + 5% CO_2_, with appropriate controls. Non-adherent bacteria were aspirated and PBS was added (200 µL/well) before fluorescence was measured. The percentage of adhered bacteria was calculated from the mean fluorescence of test wells compared to the positive control (100%).

### 3.10. Statistical Analysis

Student’s *t*-test, two-way ANOVA with Bonferroni correction and unpaired two-samples Wilcoxon test were calculated using STATISTICA 12.0 (StatSoft Polska Sp. z o.o., Kraków, Poland) software, with *p* ≤ 0.05 considered significant. Data are mostly given as means with S.D.

## 4. Conclusions

*P. officinalis* L. extract (POE; phenolic-rich fraction) was found in vitro to be an anti-staphylococcal product in terms of the inhibition of microbial adhesion, sortase A activity and α-toxin production. Such an action may be beneficial in CF patients with the initial colonization of respiratory tract by staphylococci. Rosmarinic acid (RA), being the dominant component of tested POE fraction, might be responsible for its positive antimicrobial activity, so RA-containing POE-derived products can be, like genistein, resveratrol and curcumin, a candidate for a standardized herbal supplement used for pro-health purposes in CF patients. However, the final conclusion from our studies is clear: usage of POE-containing products in prophylaxis in CF or other patients to stop the development of *S. aureus* colonization into active infection—yes; treatment/support of symptomatic, especially biofilm-associated *S. aureus* infection—certainly not.

## Figures and Tables

**Table 1:** The effect of POE used at 0.5× and 0.25 × MIC on α-toxin (Hla) production by *S. aureus* reference and clinical strains (n = 2 + 4) tested by ELISA.

*S. aureus* Strain	Hla ± S.D. [ng/mL]		
	Control (Untreated)	POE 0.5 × MIC	POE 0.25 × MIC
ATCC 29213	1035.0 ± 12.0	520.5 ± 60.7 *	632.6 ± 80.6 *
ATCC 700699	103.9 ± 8.9	60.7 ± 9.1 *	76.5 ± 12.1 *
α4	905.0 ± 70.5	839.4 ± 39.9	901.1 ± 18.1
α28	736.7 ± 12.1	426.1 ± 21.3 *	529.8 ± 40.8 *
γ14	1053.0 ± 181.9	526.5 ± 53.7 *	518.7 ± 48.2 *
γ18	109.6 ± 1.1	44.9 ± 6.3 *	93.9 ± 4.4 *

## Supplementary Materials

The following are available online, Table S1. Phytochemical composition of the examined fractions isolated from *P. officinalis* L. extract. Table S2. Metabolite content in 50% methanolic fraction of *P. officinalis* L. extract. Figure S1. UHPLC profile of *P. officinalis* extract—50% methanolic fraction (upper panel—MS base peak chromatogram using negative mode ESI, lower panel—signal form charged aerosol detector), numbers indicate compounds that were characterized and quantitatively analyzed, IS-internal standard.
