# Supplementary material for: The Pros and Cons of Cystic Fibrosis (CF) Patient Use of Herbal Supplements Containing Pulmonaria officinalis L. Extract: the Evidence from an In Vitro Study on Staphylococcus aureus CF Clinical Isolates
Source: Molecules. 2019 Mar 22;24(6):1151. doi: 10.3390/molecules24061151 (PMC6471470; doi:10.3390/molecules24061151)
Supplement: Supplementary file 1 [file molecules-24-01151-s001.pdf]

The *Pros* and *Cons* of Cystic Fibrosis (CF) Patient Use of Herbal Supplements Containing *Pulmonaria officinalis* L. Extract: the Evidence from an In Vitro Study on *Staphylococcus aureus* CF Clinical Isolates

Beata Sadowska[1], Urszula Wójcik[1], Justyna Krzyżanowska-Kowalczyk[2], Mariusz Kowalczyk[2], Anna Stochmal[b2], Joanna Rywaniak[1], Julia Burzyńska[1], Barbara Różalska[1*]

[1]Department of Immunology and Infectious Biology, Institute of Microbiology, Biotechnology and Immunology, Faculty of Biology and Environmental Protection, University of Lodz, Banacha 12/16, 90-237 Lodz, Poland
[2]Department of Biochemistry, Institute of Soil Science and Plant Cultivation, State Research Institute, Czartoryskich 8, 24-100 Pulawy, Poland

1. Plant material

Pulmonaria officinalis L. aerial parts were purchased from local herb supplier (Kania, Częstochowa, Poland). The voucher specimen (POFF./EXTR/2013/1) has been deposited at the Department of Biochemistry and Crop Quality, Institute of Soil Science and Plant Cultivation, Pulawy, Poland.

2. Preparation of lungwort methanolic fraction

Plant material was finely milled in a laboratory grinder (Retsch ZM200, Germany) and defatted with chloroform in a Soxhlet apparatus. Subsequently, defatted material (200 g) was extracted twice with 80% aq. MeOH (*v*/*v*), at room temperature for 24 h using the ultrasonic bath. The obtained extracts were filtered, supernatants were concentrated under reduced pressure and lyophilized to yield a dark brown residue (48.85 g). Crude lungwort methanol extract was further fractionated using solid phase extraction. The extract was suspended in 1% MeOH (*v*/*v*) and loaded on the preconditioned RP-C_{18} column (80×100mm, Cosmosil 140C_{18}-PREP, 140 μm; Nacalai Tesque, INC., Kyoto, Japan). 1% MeOH was also applied to remove polar constituents, while rich in phenolics fraction was eluted using 50% MeOH (*v*/*v*). Methanol was then removed under reduced pressure, and the residual material was freeze-dried yielding 6.5 g of solid.

3. Quantitative analysis of methanolic fraction from *P. officinalis*

Quantitative determination of metabolites in the examined methanolic fraction were estimated as described previously [1]. Briefly, analyses were performed on Thermo Scientific Ultimate 3000 RS chromatographic system hyphenated to a Bruker Impact II HD (Bruker, Billerica, USA) quadrupole-time of flight (TOF) spectrometer. Chromatographic separations were carried out on a Waters CORTECS T3 column (2.1×150 mm, 2.7 μm, Milford, MA, USA), using deionized water and acetonitrile containing formic acid as mobile phases. Linear (centroid) spectra were acquired over a mass range from *m*/*z* 50 to *m*/*z* 2000 with the following parameters of mass spectrometer: capillary voltage 3.0 kV; dry gas flow 6 L/min; dry gas temperature 200°C; nebulizer pressure 0.7 bar; collision RF 700.0 V, transfer time 90 μs; prepulse storage 7.0 μs. The acquired data were calibrated internally with sodium formate introduced to the ion source via a 20 μL loop at the beginning of each separation.

Chromatograms for deprotonated ions corresponding to previously identified compounds were extracted from the full scan data with a 0.005 Da width. Peaks matching the retention time and mass spectral properties of isolated standards were integrated following smoothing using the Savitzky-Golay algorithm (window width 5 points, one iteration). Ratios between the analyte peak area and the IS peak area were used for calculations. Calibration curves in the range from 0.05 to 50 μg/mL were prepared from the 1 mg/mL methanolic stock solutions of 36 investigated compounds and analyzed in the conditions specified above. Data processing was performed using Bruker DataAnalysis 4.4 SR1 software.

4. Results and Discussion

Our investigation indicated that 50% MeOH fraction from commercially available *Pulmonariae Herba* contained several known phenolic derivatives with well-documented biological properties as well as a few new compounds whose activity has yet to be investigated. Spectroscopic analyses showed that derivatives of phenolic acids, primarily conjugates of caffeic acid with danshensu, such as rosmarinic, monardic, lithospermic, salvianolic, shimobashiric and yunnaneic acids, were the most dominant compounds of the extract. Also detected were esters of caffeic acid esters with quinic (so-called chlorogenic acids), threonic and glyceric acids. Three coumaroylquinic acids were also observed. Additionally, a few lignans (globoidnans A and B, pulmonariosides A and B) and several common flavonol glycosides (quercetin and kaempferol derivatives) were also identified. Examined fraction also contained a significant amount of menisdaurin, a nitrile glucoside, and traces of actinidioionoside, a megastigmane glucoside [1]. However, our research revealed traces of pyrrolizidine alkaloids (PAs) and their *N*-oxides (PNAs) in this material (J.K.K, unpublished data), which is consistent with previous studies on a related species, *P. obscura* Dumort [2]. In the case of both *Pulmonaria* species, concentrations of alkaloids were extremely low in aerial parts, and the safety *Pulmonariae Herba* usage is unlikely to be affected by their presence. The primary organs in which PAs accumulated in higher concentrations were rhizomes and roots of lungwort plants, which are not used in phytotherapy. Also, the contents of alkaloids in *P. officinalis* is in any case significantly lower than in other plants the Boraginaceae family used in traditional medicine, such as *Borago officinalis* L. or *Symphytum officinale* L.

Phytochemical profile of the investigated fraction is shown in **Figure S1.** The estimated content of individual groups of specific metabolites is presented in **Table S1**, whereas the results of quantitative measurements are shown in **Table S2**.

Table S1. Phytochemical composition of the examined fractions isolated from *P. officinalis* L. extract.

**Content of phytochemicals (% of the fraction)**	
Phenolic acids	78.94
Lignans	9.22
Flavonol glycosides	6.72
Others	5.12

Table S2. Metabolite content in 50% methanolic fraction of *P. officinalis* L. extract.

No.	Compound name	Contents [µg/mg of fraction] (mean ± SD, n=3)
1	Danshensu	0.53 ± 0.05
2	Menisdaurin	37.83 ± 2.24
3	3-*O*-(*E*)-caffeoyl- threonic acid	1.43 ± 0.09
4	2-*O*-(*E*)-caffeoyl-L-threonic acid	2.76 ± 0.13
5	Lycoperodine-1	2.94 ± 0.2
6	Chlorogenic acid	5.18 ± 0.35
7	Actinidioionoside	Traces
8	Caffeic acid	Traces
9	Cryptochlorogenic acid	0.28 ± 0.01
10	3'-*O*-(*E*)-feruloyl-α-sorbopyranosyl-(2'→1)-α-glucopyranoside	0.44 ± 0.03
11	2-*O*-(*E*)-caffeoyl-D-glyceric acid	3.85 ± 0.17
12	4-*O*-(*E*)-caffeoyl-L-threonic acid	2.97 ± 0.11
13	Neochlorogenic acid	0.04 ± 0.00
14	3-*O*-(*E*)-caffeoyl- glyceric acid	1.86 ± 0.11
15	3-*O*-*p*-coumaroylquinic acid	3.88 ± 0.09
16	4-*O*-*p*-coumaroylquinic acid	Traces
17	5-*O*-*p*-coumaroylquinic acid	2.18 ± 0.13
18	Globoidnan B	45.06 ± 3.88
19	Rutin	1.59 ± 0.09
20	Nicotiflorin isomer	Traces
21	Quercetin 3-*O*-β-glucoside	4.03 ± 0.18
22	Yunnaneic acid E	1.81 ± 0.06
23	Quercetin 3-*O*-(6''-*O*-malonyl)-β-glucoside	9.66 ± 0.29
24	Nicotiflorin	Traces
25	Astragalin	0.87 ± 0.05
26	Shimobashiric acid C	6.72 ± 0.29
27	Rosmarinic acid	262.09 ± 21.26
28	Kaempferol 3-*O*-(6''-*O*-malonyl)-β-glucoside	1.65 ± 0.16
29	Monardic acid A	16.85 ± 1.25
30	Yunnaneic acid E-1	Traces
31	Lithospermic acid A	55.29 ± 2.89
32	Pulmonarioside A	0.13 ± 0.05
33	Salvianolic acid H	53.65 ± 2.48
34	Lithospermic acid B	NA
35	Pulmonarioside B	3.05 ± 0.15
36	Yunnaneic acid B	15.74 ± 0.92
37	Globoidnan A	1.18 ± 0.05
38	Pulmitric acid A	0.08 ± 0.01
39	Pulmitric acid B	0.05 ± 0.02
40	Isosalvianolic acid A	Traces
41	Isosalvianolic acid A-1	Traces
42	Isosalvianolic acid A isomer	Traces
43	Rosmarinic acid methyl ester	Traces
44	Salvianolic acid H-9''-methylester	5.17 ± 0.30
45	Lycopic acid C	NA

TR – traces, indicates level below the limit of quantification, NA – not analysed, ND – not detected

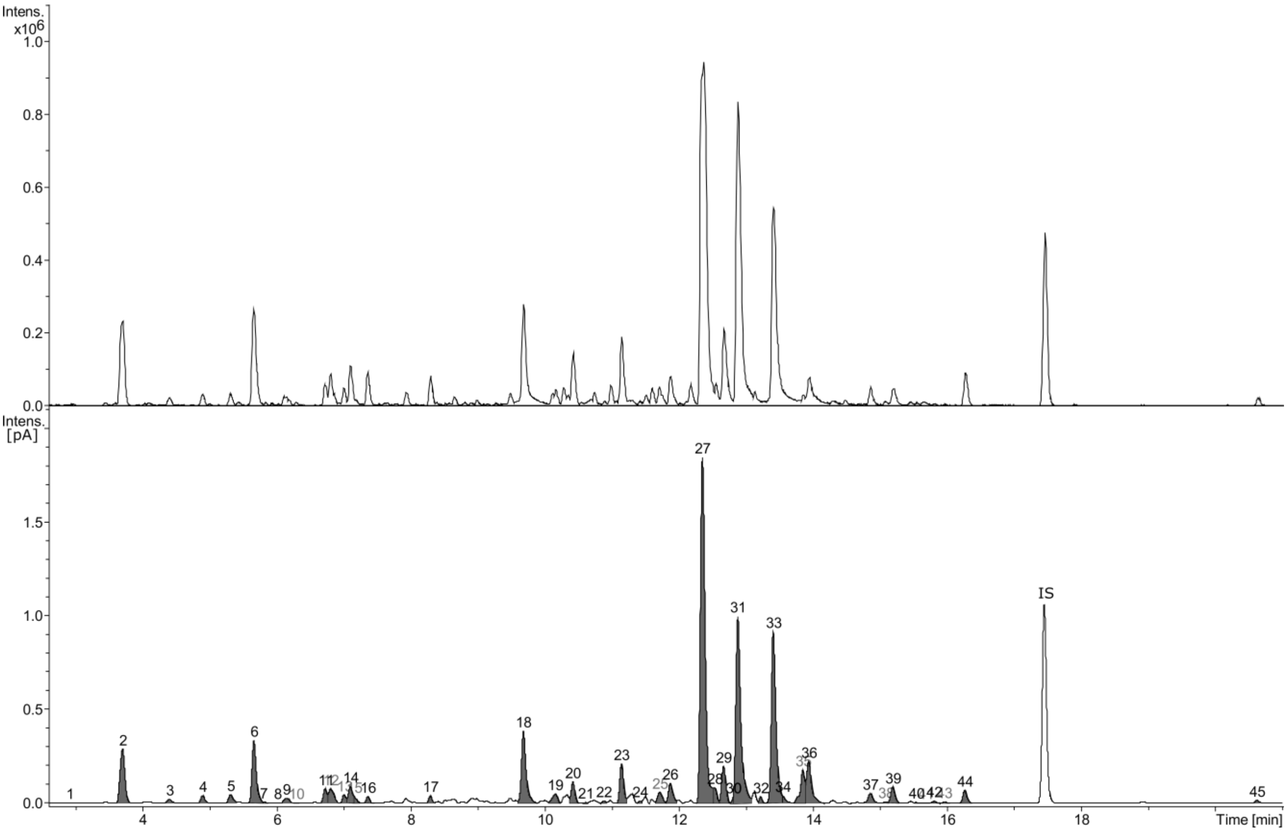

Figure S1. UHPLC profile of *P. officinalis* L. 50% methanolic fraction (upper panel – MS base peak chromatogram using negative mode ESI, lower panel – signal form charged aerosol detector, numbers indicate compounds that were characterized and quantitatively analyzed, IS-internal standard.

References

1. Krzyżanowska-Kowalczyk, J.; Pecio, Ł.; Mołdoch, J.; Ludwiczuk, A.; Kowalczyk, M. 2018. Novel phenolic constituents of *Pulmonaria officinalis* L. LC-MS/MS comparison of spring and autumn metabolite profiles. Molecules 23, 2277.
2. Haberer, W.; Witte, L.; Hartmann, T.; Dobler, S. 2002. Pyrrolizidine alkaloids in *Pulmonaria obscura*. Planta Med. 68, 480–482.
